# Ethoxy Groups on ZrO_2_, CuO, CuO/ZrO_2_ Al_2_O_3_, Ga_2_O_3_, SiO_2_ and NiO: Formation and Reactivity

**DOI:** 10.3390/molecules28083463

**Published:** 2023-04-14

**Authors:** Jerzy Podobiński, Małgorzata Zimowska, Katarzyna Samson, Michał Śliwa, Jerzy Datka

**Affiliations:** Jerzy Haber Institute of Catalysis and Surface Chemistry, Polish Academy of Sciences, Niezapominajek 8, 30-239 Krakow, Poland; jerzy.podobinski@ikifp.edu.pl (J.P.); nczimows@cyf-kr.edu.pl (M.Z.); katarzyna.samson@ikifp.edu.pl (K.S.); michal.sliwa@ikifp.edu.pl (M.Ś.)

**Keywords:** IR spectroscopy, ethoxy groups, acetate ions

## Abstract

The reaction of ethanol with surface OH groups on ZrO_2_, CuO/ZrO_2_, CuO, Al_2_O_3_, Ga_2_O_3_, NiO, and SiO_2_ was studied by IR spectroscopy. The basicity of oxides was followed by CO_2_ adsorption, and their ability to oxidize was investigated by H_2_-TPR. It has been found that ethanol reacts with surface OH groups forming ethoxy groups and water. Some oxides: ZrO_2_, CuO/ZrO_2_, Al_2_O_3_, and Ga_2_O_3_ contain several kinds of OH groups (terminal, bidentate, and tridentate) and terminal hydroxyls react with ethanol in the first order. Two kinds of ethoxyls are formed on these oxides: monodental and bidental ones. On the other hand, only one kind of ethoxy group is formed on CuO and NiO. The amount of ethoxy groups correlates with the basicity of oxides. The biggest amount of ethoxyls is produced on the most basic: ZrO_2_, CuO/ZrO_2_, and Al_2_O_3_, whereas the smallest amount of ethoxyls is produced on CuO, NiO, and Ga_2_O_3_, i.e., on oxides of lower basicity. SiO_2_ does not form ethoxy groups. Above 370 K ethoxy groups on CuO/ZrO_2_, CuO, and NiO are oxidized to acetate ions. The ability of oxides to oxidize ethoxyl groups increases in the order NiO < CuO < CuO/ZrO_2_. The temperature of the peak in the H_2_-TPR diagram decreases in the same order.

## 1. Introduction

Currently, the global economy relies on the large-scale burning of fossil fuels, which leads to environmental degradation and an uncertain future. The transition from an economy based on fossil fuels to a hydrogen-based one is commonly accepted as one of the best options to limit the emission of huge amounts of pollutants into the atmosphere. Nevertheless, the production of hydrogen, which is a promising fuel for the future, is a great challenge for science and technology. One of the feasible methods of hydrogen production is the conversion of alcohols, among which ethanol plays a crucial role. This is due to the fact that ethanol is non-toxic and can be easily produced from biomass [1,2,3,4]. On top of that, it can be easily handled and transported, which makes it a promising substrate for hydrogen production.

Hydrogen can be produced from ethanol via three main reactions: steam reforming, partial oxidation, and oxidative steam reforming. Among these methods, steam reforming is the most efficient in terms of hydrogen yield [5]. When complete steam reforming of ethanol (SRE) reaction is performed, six moles of hydrogen are produced from one mole of ethanol. This is the highest hydrogen yield it is possible to reach when compared with steam reforming of other fuels [6,7].

The nobles metals (Rh [8,9], Ru [10], Ir [11], Pd [12], Pt [13]) supported on various oxides such as ZrO_2_, Al_2_O_3_, MgO, and CeO_2_ have shown high activity towards ethanol steam reforming. Nevertheless, the high cost of noble-based catalysts seriously limits their application as catalysts for SRE [14]. Therefore, the less expensive, alternative catalysts for steam reforming are being studied. Among them, non-noble metals seem to be the right choice. Different catalytic systems based on these metals (Co, Ni, Cu) have turned out to be active for SRE reactions [15,16,17,18]. Copper-based catalysts are mainly used in methanol steam reforming due to their high selectivity and activity [19,20], but there are also studies on the application of copper-containing catalysts in the steam reforming of ethanol. Galetti et al. [21] investigated the steam reforming of ethanol over quaternary mixed oxide CuCoZnAl catalyst.

Our previous studies regarding SRE over CuO/ZrO_2_ modified with ZnO, Ga_2_O_2_, NiO, and MnO oxides show that these catalysts are active for ethanol conversion toward hydrogen production at 350 °C. It was proved that basic sites are responsible for acetaldehyde formation and the addition of NiO improves the catalyst’s ability to C-C bond cleavage. Moreover, our studies on the deactivation of catalysts in SRE by means of XPS, TPO, and Raman spectroscopy revealed that the observed decrease in hydrogen yield for catalysts containing ZnO is due to the carbon deposition formation and adsorption of organic by-product on the catalysts’ surface [7,22].

Since the formation of ethoxyl groups is considered to be the first step of ethanol conversion, the goal of our scientific work was to elucidate and describe the process of ethoxyl group formation on different oxides, i.e., CeO_2_ [23], ZrO_2_, CuO, and CuO/ZrO_2_ [24], and further transformation of these groups in consecutive reactions, which was followed by FTIR spectroscopy. It was found that mono-, di-, and tridentate ethoxyls were formed on CeO_2_, showing various susceptibilities for oxidation. Among detected ethoxyls groups on CeO_2_, monedetate ethoxyls were the first to be oxidized by CeO_2_, whereas tridentate species were the last ones. In the case of ZrO_2_, CuO, and CuO/ZrO_2_, ethoxy groups were also formed but the chemical pathways of their transformations were different, depending on the type of investigated oxide. At higher temperatures, ethoxy groups on ZrO_2_ were transformed to ethene [24]. On the other hand, ethoxy groups were oxidized to acetate ions without the formation of acetaldehyde when CuO and CuO/ZrO_2_ were considered. It is worth noting that acetaldehyde was formed on these oxides only if gaseous ethanol was in the cell.

The aim of these studies, which are a continuation of our previous findings, was to investigate the possible chemical reaction pathways of ethanol transformation over ZrO_2_, CuO, CuO/ZrO_2_, NiO, and Ga_2_O_3_ oxides being used as components of steam reforming catalysts. Additionally, we also investigated Al_2_O_3_ and SiO_2_. We paid special attention to elucidate the role of surface hydroxyls and surface basicity in the formation of ethoxy groups. Another interesting point of our study was to investigate the effect of the oxidative properties of oxides on the process of the formation of acetate ions. Having all the above in mind, we decided to choose oxides of various basicity and different oxidative properties.

Such a complex study of ethanol transformations on ZrO_2_, CuO, CuO/ZrO_2_, NiO, and Al_2_O_3_ and Ga_2_O_3_ have not been undertaken before, since more attention was paid to investigate methanol reactions. Neither the effect of surface basicity nor the effect of oxidative properties of oxides on acetate ions formation were discussed before. The formation of ethoxy groups and their oxidation to acetate ions was followed by IR spectroscopy. Their basicity was studied by CO_2_ adsorption and the oxidative properties of oxides were followed by temperature programed reduction (H_2_-TPR).

## 2. Results and Discussion

### 2.1. Structure and Morphology

**Figure 1:**
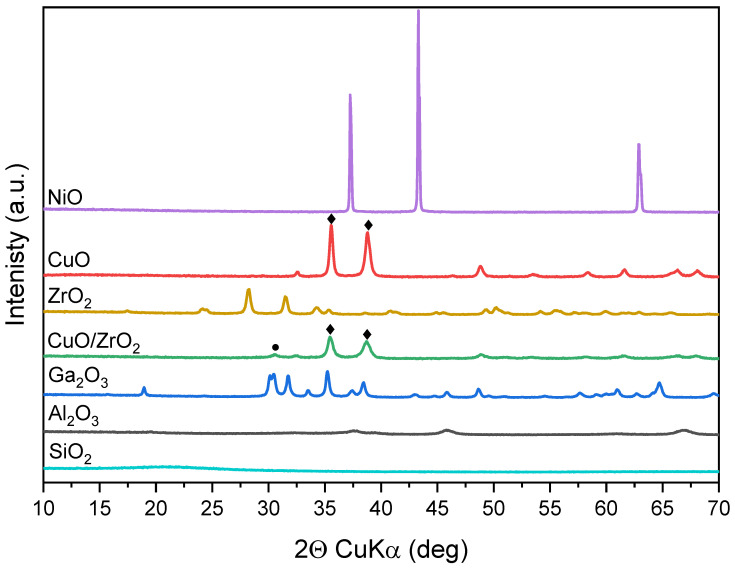
XRD patterns of the oxides. CuO (♦), ZrO_2_ (●).

To evaluate the crystal structure of the oxides, all samples were analyzed with the use of the XRD method. Figure 1 shows the XRD patterns with the phase composition and crystallinity of the samples. The diffractograms of amphoteric Al_2_O_3_ and SiO_2_ oxides exhibit the most amorphous character, while the Ga_2_O_3_ shows the presence of intense reflections assigned to the planes crystalized in C2/m monoclinic symmetry. The diffractograms of CuO and ZrO_2_ display reflections due to the presence of well-ordered monoclinic phases crystallized in the C2/c and P21/c symmetry [ICDD PDF-4+ 2015 04-004-4916] and [ICDD PDF-4+ 2015 00-036-0420], respectively. NiO sample crystallizing within cubic Fm-3m symmetry exhibits the most intensive reflections, with the average crystallite dimension around 40–70 nm [ICDD PDF-4+ 2015 04-007-5695]. The sample CuO/ZrO_2_ is a mixture of two oxides of lower crystallinity with reflections attributed to CuO of the monoclinic phase confirmed by Bragg peaks at 35.48 and 38.71° of 2θ, and ZrO_2_ crystallized in cubic space Fm-3m group indicated by the presence of reflection at 30.56° of 2θ [ICDD PDF-4+ 2015 04-003-2609].

Detailed analysis of XRD patterns by the Scherrer method indicates that the crystallite sizes of CuO and ZrO_2_ and Ga_2_O_3_ oxides are within the range of 20–40 nm, while crystallites of Al_2_O_3_ are lower than 10 nm in the studied samples. Based on the calculation, we have found that the crystallite dimensions of the synthesized CuO/ZrO_2_ mixture are in the same range of 10–20 nm.

**Figure 2:**
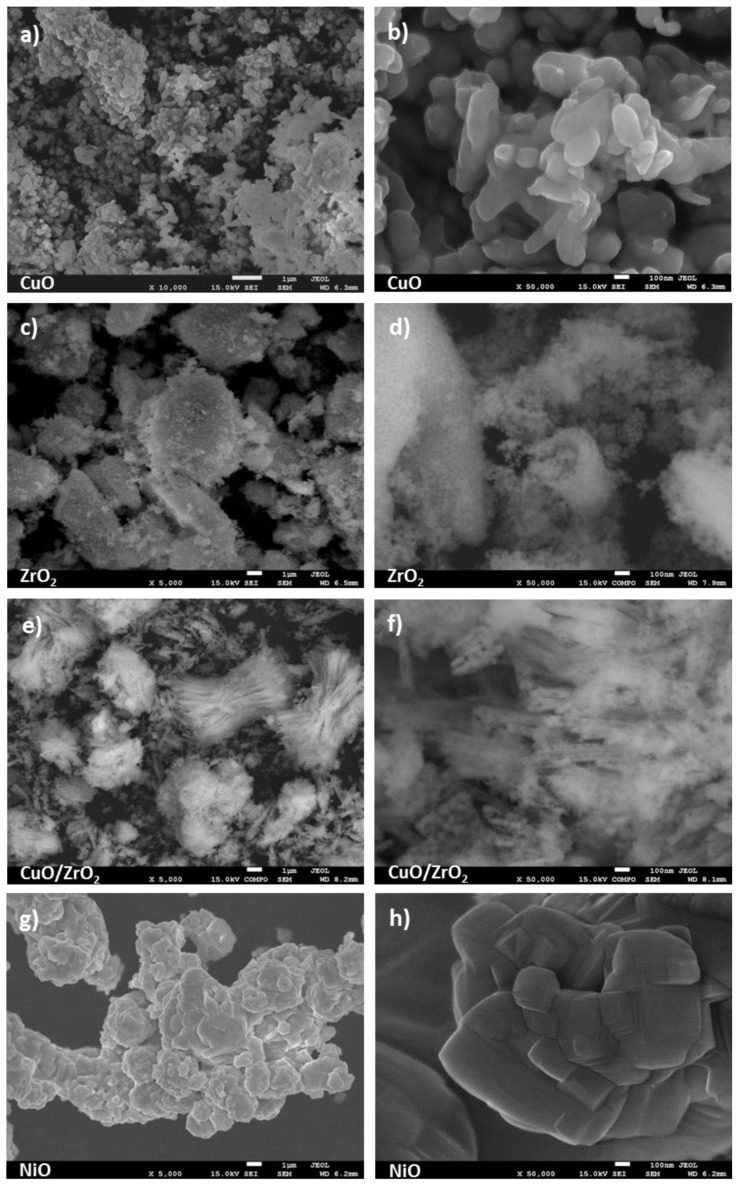
SEM images of oxides: (**a**,**b**) CuO; (**c**,**d**) ZrO_2_; (**e**,**f**) CuO/ZrO_2;_ (**g**,**h**) NiO samples.

**Figure 3:**
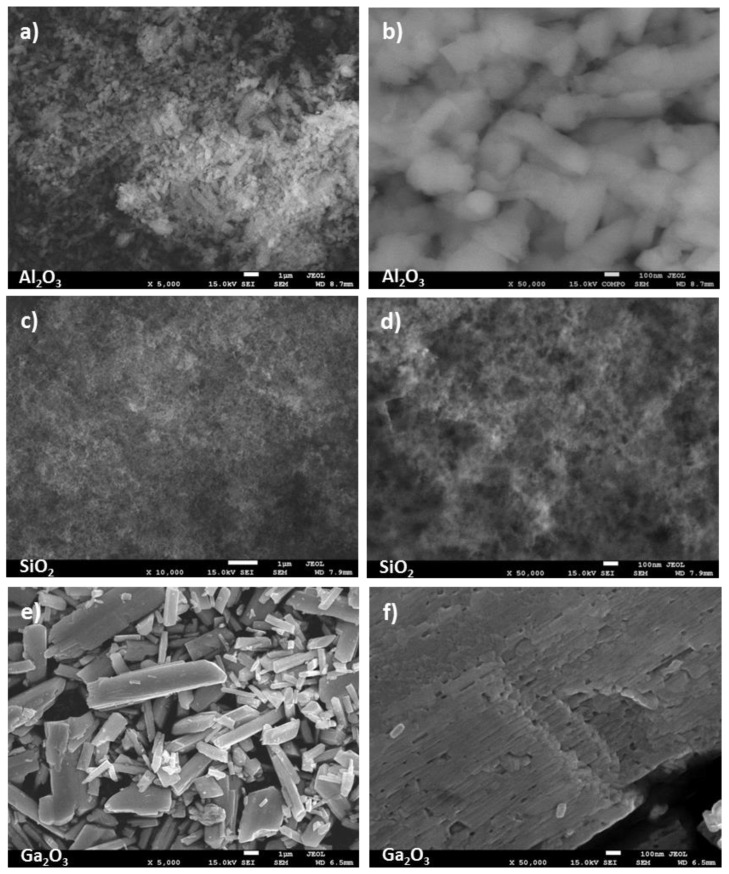
SEM images of oxides: (**a**,**b**) Al_2_O_3_; (**c**,**d**) SiO_2_; (**e**,**f**) Ga_2_O_3_ samples.

In Figure 2 and Figure 3, the secondary electron images of the oxides are presented. They clearly illustrate the differences in the morphology of the examined samples. SEM analysis reveals that, generally, oxides are composed of fine particles, and only CuO and NiO form bigger, more crystalline particles (Figure 2b,h). It is worth noticing that the Ga_2_O_3_ sample exhibits the presence of 1–8 μm grains but is composed of crystallites not exceeding 100 nm, which is in line with our XRD calculation (Figure 2f). A very interesting morphology shows synthesized CuO/ZrO_2_ mixed oxides forming the secondary structure of spindles, whose long fibers are made of very tiny 10–20 nm crystallites well visible in Figure 2f.

### 2.2. Hydroxyl Groups

**Figure 4:**
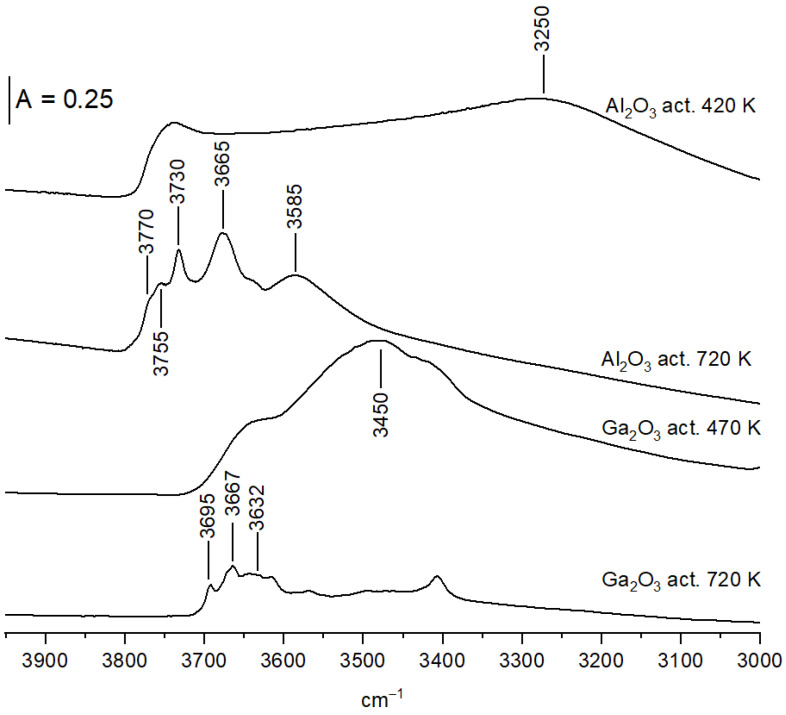
The IR spectra of OH groups on Al_2_O_3_ and Ga_2_O_3_ activated in vacuum at 470 and 720 K.

In order to remove the physisorbed water and other molecules, the wafers of oxides were pretreated in a vacuum at 470 K. These applied conditions were sufficient to remove water, which was evidenced by the disappearance of the 1640 cm^−1^ band. For two oxides, Al_2_O_3_ and Ga_2_O_3_, strong bands of oxo-hydroxo species (3250 and 3450 cm^−1^ resp.) were present [23,25,26]—Figure 4 and overlapped Al-OH and Ga-OH bands. These two oxides were pretreated in a vacuum at the higher temperature of 820 K. The bands of oxo-hydroxo species diminished significantly upon pretreatment at 820 K, and distinct bands of surface OH groups are visible (Figure 4).

**Figure 5:**
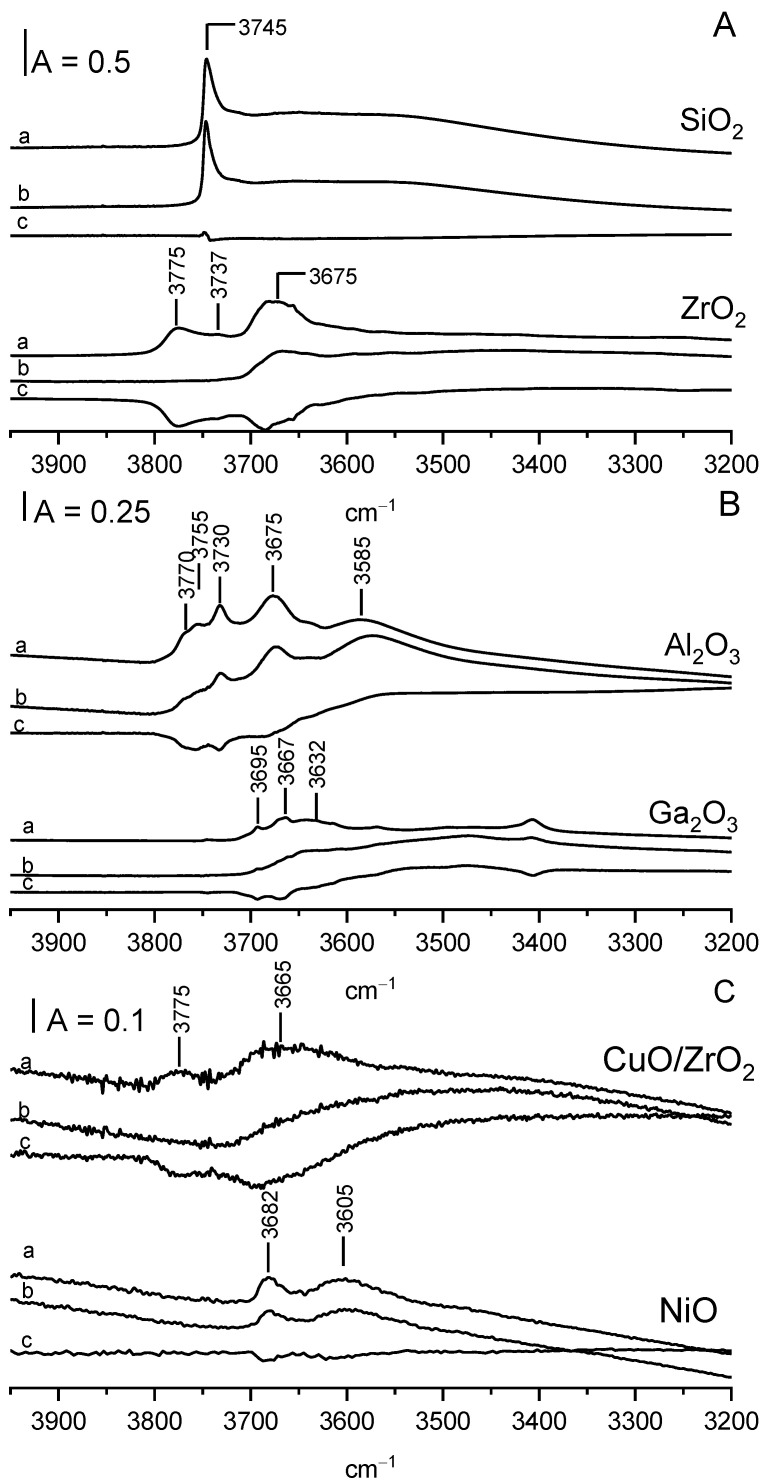
(**A**)—OH groups on SiO_2_ and ZrO_2_ activated (a), upon adsorption of ethanol at room temperature followed by evacuation at 370 K (b) and difference spectrum (c = b − a). (**B**)—OH groups on Al_2_O_3_ and Ga_2_O_3_ activated (a), upon adsorption of ethanol at room temperature followed by evacuation at 370 K (b) and difference spectrum (c = b − a). (**C**)—OH groups on CuO/ZrO_2_ and NiO activated (a), upon adsorption of ethanol at room temperature followed by evacuation at 370 K (b), and difference spectrum (c = b − a).

The frequencies of the OH bands in our oxides are given in Table 1. The spectrum of SiO_2_ (Figure 5A) shows only one narrow and intensive band of surface Si-OH groups at 3746 cm^−1^.

The spectrum of OH groups on the ZrO_2_ surface (Figure 5A) shows three distinct OH bands at 3675, 3737, and 3775 cm^−1^. The same OH bands were also reported by other authors [26,27,28,29,30,31] and assigned to tribridged (3675 cm^−1^), dibridged (3637 cm^−1^), and terminal Zr-OH (3775 cm^−1^). According to the literature [30], terminal hydroxyls are single cations at oxygen lattice faces, whereas the multi-coordinated hydroxyls are located at low index faces.

The spectrum of OH groups on Al_2_O_3_ (Figure 5B) shows five distinct OH bands at 3585, 3675, 3730, 3755, and 3770 cm^−1^. The spectra of Al-OH groups were analyzed by several authors [32,33,34], and the revue paper on this subject was conducted by Knozinger and Ratnasamy [35]. According to these authors (similar for ZrO_2_), monobridged (terminal), bridged, and tribridged hydroxyls are present in Al_2_O_3_. The band at 3770 cm^−1^ can be assigned to terminal Al-OH in which Al is four-coordinated, 3755 cm^−1^—to bridged hydroxyls in which both Al are hexa-coordinated, 3730 cm^−1^—to bridged hydroxyls in which one Al is four-coordinated and the second one is hexa-coordinated. A 3675 cm^−1^ band was assigned to tribridged hydroxyls in which all three Al are hexa-coordinates. The band at 3585 cm^−1^ may be due to oxohydroxy species. These free OH groups were denoted as type I, IIB, IIA, and III. According to Knozinger et al. [35], they differ in the electrical charge: those of type I are the most negative, and those of type III are positive.

The spectrum of OH groups on Ga_2_O_3_ (Figure 5B) also shows several maxima. Three bands can be distinguished: 3695, 3667, and a broad band at 3632 cm^−1^. Similar OH bands were reported by Otero-Arean et al. [36], who denoted them (by analogy to Al_2_O_3_) as type I, II, and III. Generally, the situation with OH groups on gallium oxide is similar to that on alumina. Similar types of OH are present, but the OH frequencies for gallium oxide were lower than for alumina. This phenomenon was explained [36] in terms of the higher electronegativity of Ga when compared with Al, and, therefore, a higher covalency of Ga-O bond and a weaker O-H bond.

The spectrum of OH groups on CuO/ZrO_2_ (Figure 5C) is very similar to the spectrum of OH on ZrO_2_, but OH bands are distinctly smaller.

The spectrum of dehydrated NiO shows two weak OH bands at 3682 and 3605 cm^−1^. The existence of OH groups on NiO (100) and (111) surfaces was evidenced by Cappus et al. [37] by using surface-sensitive spectroscopies. It is not excluded that two IR bands of Ni-OH correspond to the two kinds of surface hydroxyls reported by Cappus et al. [37].

The transmission of IR radiation for CuO in the OH region was so poor that the observation of IR bands of OH groups was not possible.

The reaction of surface hydroxyls with ethanol was followed by the adsorption of an excess of ethanol (ca. 10 Torr in the gas phase) at room temperature, and (after 30 min of reaction at RT) removal of physisorbed ethanol by evacuation at 370 K. The spectra recorded upon such a treatment (spectra b in Figure 5) show the decrease in the bands of the OH groups. The difference spectra (spectra c—spectra recorded upon adsorption minus spectra before adsorption) are also shown. Only in the case of SiO_2_, surface Si-OH groups did not react with ethanol at room temperature. The differential spectrum practically did not show a loss of Si-OH. In all other cases (for ZrO_2_, CuO/ZrO_2_, Al_2_O_3_, Ga_2_O_3_, and NiO), the reaction of ethanol with surface hydroxyls caused some decrease in OH bands (seen clearly in differential spectra in Figure 5).

The analysis of the spectra presented in Figure 5 evidences that not all hydroxyl groups react with ethanol at room temperature. It is interesting to note that for ZrO_2_, Al_2_O_3,_ and Ga_2_O_3_, the high-frequency hydroxyls, i.e., OH of type I (terminal), react with ethanol at the first order, and OH III (of lowest OH frequency) react at the last order. This order can be explained assuming (according to Knozinger et al. [35]) that terminal high-frequency hydroxyls of type I have the highest negative charge (i.e., are the most basic) and are the most prone to react with ethanol, which shows a weakly acidic character.

**Figure 6:**
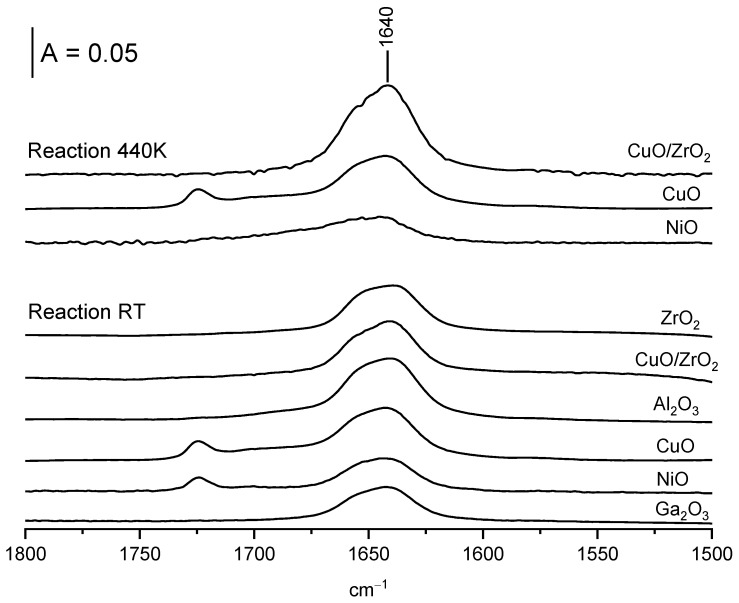
The spectra of water desorbed from oxides and readsorbed on zeolite NaY.

Two mechanisms of the formation of ethoxy groups were presented by several authors. According to one mechanism, the new OH groups were formed as the product of the reaction of alcohol with oxide: CeOCe + HOC_2_H_5_ = Ce-OH + Ce-O-C_2_H_5_. According to a second mechanism, hydroxyl groups are consumed and water is formed: Ce-OH + HOC_2_H_5_ = Ce-OC_2_H_5_ + H_2_O. Even though the consumption of surface hydroxyls (terminal, bibridged, and tribridged ones) observed in our study suggests the second mechanism, we realized experiments, the goal of which was to check whether water was indeed formed. The dose of ethanol (ca. 10 µmol) was adsorbed at room temperature on all the oxides inside the IR cell, and the products of the reaction were desorbed to cold trap and subsequently readsorbed on the wafer of zeolite NaY pretreated at 670 K. The IR spectra of zeolite with the adsorbed products of the reaction of ethanol with the oxides are presented in Figure 6. All the spectra show a 1640 cm^−1^ band typical of molecular water, providing evidence that the reaction of ethanol with hydroxyl groups on all the oxides proceeds according to the second mechanism. In our recent study [23], we followed the reaction of surface OH groups on CeO_2_ and evidenced that the first doses of ethanol reacted with CeO_2_ according to the first mechanism (i.e., new hydroxyls were formed and water was not produced), whereas only the subsequent ethanol doses reacted according to the second mechanism. The obtained results in this study evidence the second mechanism of ethanol reaction for all the oxides studied.

### 2.3. Ethoxy Groups on Oxides

The interpretation of the IR spectra of ethoxy groups is more difficult than for methoxy groups because the vibration of C-C-O entities gives three IR bands of symmetric, asymmetric stretching (around 900 and 1050 cm^−1^) as well as combination band (around 1100 cm^−1^).

As said above, the reaction of ethanol with Si-OH at room temperature does not form ethoxy groups. There is no loss of OH groups, and no bands around 1000 cm^−1^ are present.

**Figure 7:**
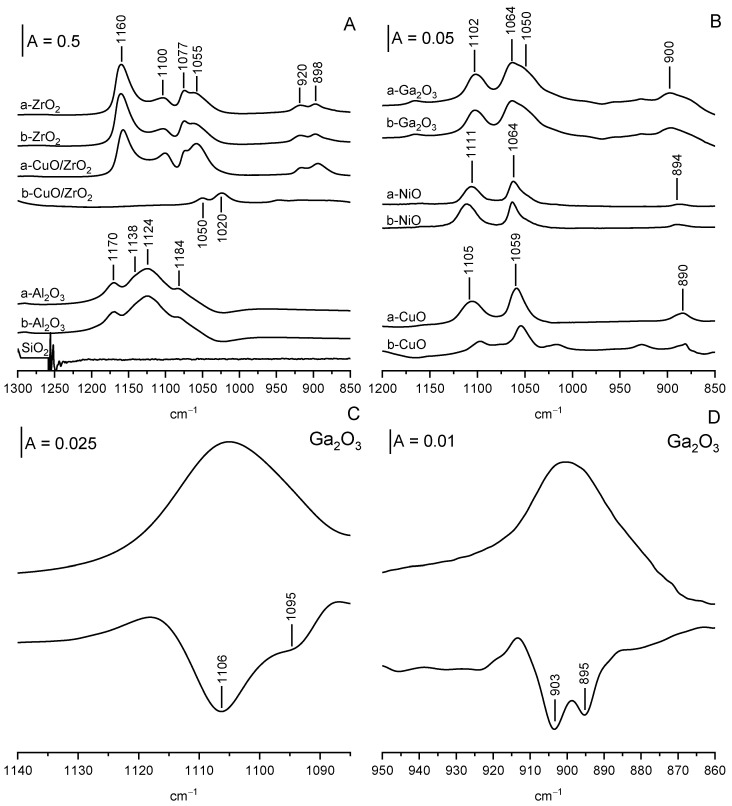
Ethoxy groups on ZrO_2_, CuO/ZrO_2_, on Al_2_O_3_ (**A**), and on Ga_2_O_3_, NiO, and CuO (**B**). Spectra were recorded upon adsorption of excess of ethanol (ca. 10 Torr in gas phase) followed by evacuation at 370 K (a) and upon heating in closed cell to 440 K (b). The spectra of ethoxy groups on Ga_2_O_3_ in the region ca 1100 (**C**) and ca, 900 cm^−1^ (**D**) and the second derivative diagrams.

All other oxides form ethoxy groups. The spectra of ethoxy groups are presented in Figure 7A,B. Generally, the oxides studied by us can be divided into two groups. For ZrO_2_, CuO/ZrO_2_, Al_2_O_3,_ and Ga_2_O_3_, the spectra of ethoxy groups show 5–6 maxima. For CuO and NiO, only three maxima are present. ZrO_2_ and CuO/ZrO_2_ contain Zr-OH terminal and tribridged (the contribution of dibridged OH is small). The reaction of ethanol with these hydroxyls forms monodental (1160, 1077, and 920 cm^−1^) and bidental ethoxyls (1100, 1055 and 898 cm^−1^) (Figure 7A).

Al_2_O_3_ contains surface OH of type I, II, and III. The analysis of the OH spectra presented in Figure 5B suggests that practically only type I (terminal) and II (bridged) hydroxyls react with ethanol. Therefore (by analogy with ZrO_2_), it can be suggested that two kinds of ethoxyls are present upon reaction with ethanol. The spectrum of ethoxy groups (Figure 7A) shows three distinct maxima (1170, 1124 and 1084 cm^−1^) as well as hardly noticeable shoulder at 1138 cm^−1^. The spectrum in the region around 900 cm^−1^ is illegible because of the overlapping strong band of Al-O. We suppose that the 1170 and 1024 cm^−1^ bands can be due to monodental ethoxyls, whereas 

The situation with Ga_2_O_3_ is somewhat similar to Al_2_O_3_. The analysis of OH spectra (Figure 5B) suggests that practically only OH groups of type I and type II react with ethanol at room temperature. Therefore, it may be supposed that monodental and bidental ethoxyls can be formed. The analysis of the spectra of ethoxy groups (Figure 7B) suggests that it is true. The spectrum of ethoxy groups shows three distinct maxima at 1102, 1064, and 900 cm^−1^ and a shoulder at 1050 cm^−1^. The analysis of the second derivative diagrams (Figure 7C,D) shows that 1102 and 900 cm^−1^ maxima are complex and each of them is composed of two submaxima. Therefore, all together, there are six maxima of ethoxy groups formed on Ga_2_O_3_: 1106, 1095, 1064, 1050, 903, and 895 cm^−1^. It may be supposed that by analogy to ZrO_2_ maxima at 1106, 1064, and, 903, cm^−1^ can be due to monodentate and 1095, 1050, 895 cm^−1^ to bidentate ethoxyls.

The spectra of ethoxy groups formed on CuO and NiO are presented in Figure 7B. For each oxide, three maxima are present. For NiO, the maxima of ethoxy groups appear at 1111, 1064, and 894 cm^−1^. For CuO, the maxima at 1105, 1059, and 890 cm^−1^ appear. It can be concluded that for each of these oxides, only one kind of ethoxy group is formed. It is difficult to decide if they are monodendate of bidendate ones, although the comparison of the band positions of ethoxyls on CuO and NiO with those on ZrO_2_ suggests that bidendate ethoxyls were formed on CuO and NiO. The frequencies of IR bands of ethoxy groups formed on oxides are given in Table 1.

In summary, it can be said that for some oxides: ZrO_2_, CuO/ZrO_2_, Al_2_O_3,_ and Ga_2_O_3_, two kinds (monodental and bidental) of ethoxyl groups are formed by the reaction of ethanol with surface hydroxyls. On the other hand, for NiO and CuO, only one kind of ethoxy group is formed.

### 2.4. Correlation of Amount of Ethoxy Groups with Basicity of Oxides

According to the data presented in Figure 5, the reaction of ethanol-producing ethoxy groups consumes some hydroxyls. All the oxides, which form ethoxy groups, are oxides of metals (Zr Al, Ga, Cu, Ni), of which surface hydroxyls have the basic character. Therefore, we correlated the number of ethoxy groups (expressed as A_370_/m, where A_370_ is the integrated intensity of IR bands of ethoxy groups) with the basicity (B/m, where B is the amount of CO_2_ adsorbed). CO_2_ was adsorbed by the pulse method, and the details on the method are given in the Materials and Methods section. The amounts of CO_2_ adsorbed as well as the intensity of ethoxy groups bands are presented in Table 2. The basicity is the product of the multiplication of the surface area (in m^2^/g) and the “specific basicity” (number of sites/m^2^). Generally, our oxides can be divided into two groups. The oxides of the first group (ZrO_2_, CuO/ZrO_2,_ and Al_2_O_3_) contain a higher number of basic sites (B/m = 44–50 µmol/g) and produce also a higher number of ethoxyls (A_370_/m = 48–50 cm^−1^/g). On the other hand, the oxides of the second group (Ga_2_O_3_, CuO, and NiO) contain a rather low number of basic sites (B/m = 0.1–5.8 µmol/g) and they produce a small amount of ethoxyls (A_370_/m = 2.0–4.7 cm^−1^/g).

Therefore, it may be concluded that the basic character of surface hydroxyls determines the ability of ethoxy group formation. On the other hand, SiO_2_, which contains a high amount of surface nonbasic Si-OH, does not form ethoxy groups.

### 2.5. Oxidation of Ethoxy Groups

**Figure 8:**
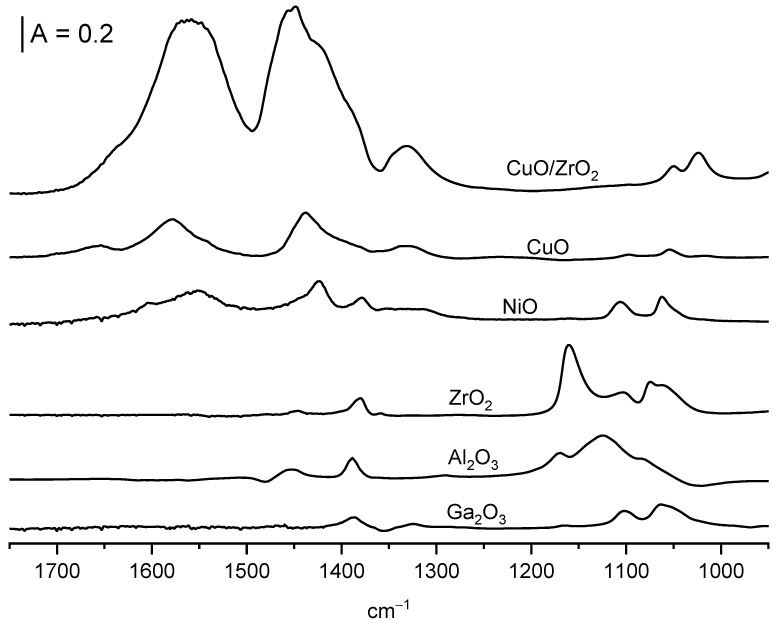
The spectra recorded upon the heating of oxides with ethoxy groups to 440 K.

When CuO, CuO/ZrO_2_ and NiO are heated to a temperature of 440 K, the ethoxyl groups, which are present on their surfaces, undergo oxidation leading to formation of acetate ions, characterized by the bands of symmetric and asymmetric stretching of COO^−^ entities at ca. 1450 and 1560 cm^−1^ (Figure 8). Similar bands of acetic ions were observed in the earlier study in which acetic acid was adsorbed on CeO_2_ [23,38] as well as in the spectra of sodium and potassium acetate [39]. The bands of ethoxy groups diminish (Figure 7 spectra b). The heating of ethoxyl groups on ZrO_2_, Al_2_O_3_, and Ga_2_O_3_ to 440 K does not produce acetate ions (Figure 8), and the intensity of IR bands of ethoxy groups practically does not change (Figure 7). Only the heating of ethoxyl groups on Al_2_O_3_ and on ZrO_2_ above 470 K produces ethene, which is the product of dehydration (spectra not shown).

The ability of CuO, CuO/ZrO_2_, and NiO to oxidate ethoxy groups was followed by comparing the values of A_440_/A_370_, where A_370_ and A_440_ are integrated intensities of the bands of ethoxy groups upon the evacuation at 370 and heating to 440 K, respectively. The lower A_440_/A_370_ is, the more ethoxy groups are oxidized, i.e., the better oxidizer is the oxide. The A_440_/A_370_ values for CuO, Cu/ZrO_2_, and NiO are presented in the Table. The A_440_/A_370_ values decrease the order NiO > CuO > CuO/ZrO_2_. The lowest value (A_440_/A_370_ = 0) for CuO/ZrO_2_ means that all the ethoxy groups were oxidized to acetate ions. The highest A_440_/A_370_ value for NiO (0.65) means that only ca. 35% of ethoxy groups have been oxidized.

**Figure 9:**
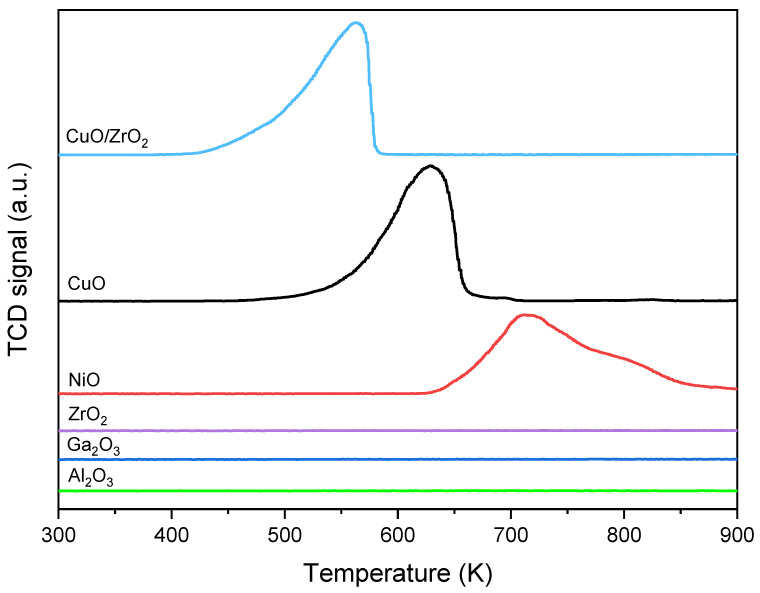
The H_2_-TPR profiles of oxides.

The ability of oxides for oxidation of ethoxy groups can be correlated with the results of hydrogen TPR presented in Figure 9. The maximum temperature on the TPR diagram is 550 K for CuO/ZrO_2_, 620 K for CuO, and 710 K for NiO (the Table). Therefore, CuO/ZrO_2_ is the most effective oxidizer for ethoxy groups (and also for hydrogen), and NiO is the less effective oxidizer. It should be noted that ZrO_2_, Al_2_O_3_, and Ga_2_O_3_, which do not produce acetate ions at 440K, are not reduced by H_2_ (Figure 9).

The oxidation of ethoxy groups to acetate ions is accompanied by the formation of water. It was evidenced in the experiments in which the ethoxy groups were formed in situ in an IR cell by adsorption of the dose of ethanol (ca. 10 µmol) at room temperature, followed by evacuation at 370 K and subsequent heating of oxide with ethoxyls in a closed cell to 440 K. The product of oxidation of ethoxyls to acetate ions was desorbed to cold trap. Next, these products were adsorbed on the dehydrated wafer of zeolite NaY. The band of water at 1640 cm^−1^ adsorbed on zeolite was present for CuO, CuO/ZrO_2,_ and NiO (Figure 6), thus evidencing that the oxidation of ethoxy groups to acetate ions is accompanied by the formation of water. It should be noted that for NiO, the amount of water formed is smaller than for CuO and CuO/ZrO_2_. This can be related to the fact that the NiO is the less effective oxidizer and that the smallest fraction of ethoxyl groups were oxidized to acetate ions. It seems probable that water is produced according to the reaction M-O-C_2_H_5_ + 2O → M^+^ + ^−^OOC-CH_3_ + H_2_O.

The situation with our oxides CuO, CuO/ZrO_2,_ and NiO was unlike that observed for CeO_2_ [23]. Oxidation of ethoxyl groups did not produce water, but new hydroxyl groups were formed.

## 3. Materials and Methods

SiO_2_ (Cab-osil M5 produced by Riedel de Haen) was used. Al_2_O_3_ (ACS reagent, >99.6% purity), Ga_2_O_3_ (ACS reagent, ≥99.99% purity), NiO (ACS reagent, 99.99% purity), and ZrO_2_ (ACS reagent 99.99 purity) were purchased from Sigma-Aldrich, St. Louis, MO, USA, whereas CuO standard (ACS reagent, ≥99.0% purity) was purchased from Merck. These oxides were used without further purification.

ZrO_2_ was synthesized by dropwise addition of 30 wt% of ammonia to the 0.5 M solution of ZrO_2_(NO_3_)_2_ · 6H_2_O at pH = 1.5. The precipitated material was heated under reflux at 370 K for 48 h. Next, the obtained product was filtered, washed with H_2_O, dried at 373 K, and calcined at 887 K.

The CuO/ZrO_2_ catalyst was synthesized via the co-precipitation method at pH = 7, using Na_2_CO_3_ as a precipitating agent. The CuO to ZrO_2_ weight ratio was fixed to 2.3. Cations in the form of nitrates and Na_2_CO_3_ were simultaneously added dropwise into the beaker containing 100 mL of deionized water at 333 K. The mixture was vigorously stirred during the precipitation. Next, the precipitate was washed by five-time centrifugation at 4200 rpm, dried at 373 K, and calcined at 823 K for 3 h. For both obtained ZrO_2_ and CuO/ZrO_2_, the yield of the synthesis was 97% and 98%, respectively, taking into account the concentration of the metal precursors in the used solutions and stoichiometry of the reaction between metal precursors and precipitation agents.

X-ray diffraction (XRD) patterns were collected with the X’Pert PRO MPD diffractometer (PANalytical, Almelo, The Netherlands) with CuKα radiation (40 kV, 30 mA) selected by a nickel monochromator in a diffraction beam with a step size 0.05°. The pattern was recorded in the range of 2–92° with the use of a silicon low background sample holder. The crystal size of the oxide was estimated using the Scherrer equation based on the fwhm (full width at half-maximum) measurement of the reflections.

The morphology of the sample was carried out by means of a JEOL JSM–7500F Field Emission Scanning Electron Microscope (JEOL, Akishima, Japan) equipped with a retractable backscattered-electron detector (RBEI) and energy dispersive spectra (EDS) detection system of a characteristic X-ray radiation Ztec Live for an EDS system (Oxford Instruments, Abingdon, UK).

Temperature-programmed reduction with hydrogen (H_2_-TPR) was carried out on a Chembet-3000 (Quantochrome, Boynton Beach, FL, USA). The hydrogen consumption was monitored with a TCD detector. For the typical H_2_-TPR experiment, the sample (25 mg) was placed in a quartz U-shape tube reactor and activated at 370 K in He flow (30 mL/min) for 1.5 h. Next, the sample was cooled down to RT in He flow and the H_2_-TPR experiment was performed in 5% H_2_/Ar (30 mL/min) in the temperature range RT–920 K (∆T = 10 K/min).

The adsorption of 5% CO_2_/Ar pulses (250 µL) at RT was used to evaluate the basicity of the study materials. Prior to adsorption, samples (50 mg) were activated in the stream of He (30 mL/min) at 450 °C for 30 min. Next, the reactor was cooled down to RT and the pulses of 5% CO_2_/Ar were introduced until saturation. The signal *m*/*z* = 44 (CO_2_) was monitored with mass spectrometer (QMS).

For IR studies, all oxides were pressed into thin wafers of ca. 100–250 mg. Prior to IR experiments, wafers were evacuated in situ in an IR cell at 470 K for 30 min. In some experiments, Al_2_O_3_ and Ga_2_O_3_ were evacuated at 720 K. Ethanol was adsorbed at room temperature and subsequently heated to various temperatures. After each heating step, the cell was cooled to room temperature and the IR spectrum was recorded. The spectra were recorded with a NICOLET 6700 spectrometer (Thermo Scientific, Cambridge, MA, USA) with a spectral resolution of 1 cm^−1^.

## 4. Conclusions

The reaction of ethanol with surface hydroxyls on ZrO_2_, CuO/ZrO_2_, CuO, Al_2_O_3_, Ga_2_O_3_, NiO, and SiO_2_ was studied by IR spectroscopy. It has been confirmed that ethanol reacts with surface OH groups of all studied oxides, except for SiO_2_, forming ethoxy groups and water. ZrO_2_, CuO/ZrO_2_, Al_2_O_3,_ and Ga_2_O_3_ contain terminal, bridged, and tribridged hydroxyls. In the case of these oxides, ethanol reacts with terminal hydroxyls in the first order, leading to formation of monodental and bidental ethoxyls. On the other hand, only one kind of ethoxyl group is formed on CuO and NiO. The number of ethoxy groups was correlated with the concentration of basic sites, which was measured by pulse CO_2_ adsorption. The highest amount of ethoxy groups was formed for oxides of the highest surface basicity, i.e., ZrO_2_, Al_2_O_3,_ and CuO/ZrO_2_. It was shown that above 370 K, ethoxy groups on CuO, NiO, and CuO/ZrO_3_ are oxidized to acetate ions and water. The ability of oxides to oxidize ethoxyl groups increases in the order: NiO < CuO < CuO/ZrO_2_. The temperature of the peak in the H_2_-TPR profiles decreases in the same order.

## Figures and Tables

**Table 1:** The frequencies of IR bands of hydroxyl groups and ethoxy groups on oxides.

Samples	Frequencies of OH Groups/cm^−1^	Frequencies of Ethoxy Groups/cm^−1^
SiO_2_	3745	-
ZrO_2_	3775, 3737, 3675	1160 (M), 1077 (M), 920 (M) 1100 (B), 1055 (B), 898 (B)
CuO/ZrO_2_	3775, 3665	1160 (M), 1077 (M), 920 (M) 1100 (B), 1055 (B), 898 (B)
Al_2_O_3_	3770, 3755, 3730, 3675, 3585	1070 (M), 1024 (M) 1138 (B), 1084 (B)
Ga_2_O_3_	3695, 3667, 3632	1106 (M), 1064 (M), 903 (M) 1095 (B), 1050 (B), 895 (B)
CuO	-	1105, 1059, 890
NiO	3682, 3605	1111, 1064, 894

M—monodental ethoxyls, B—bidental ethoxyls.

**Table 2:** The basicity of oxides (B—the amount of CO_2_ adsorbed per gram), the number of ethoxyl groups (A/m—the integrated intensity of IR bands per gram) upon evacuation at 370 K and upon reaction at 440 K. The peak temperature in H_2_-TPR profile.

Samples	B µmol CO_2_/g	A_370_/m cm^−1^/g	A_440_/m cm^−1^/g	A_440_/A_370_	Peak temp. H_2_-TPR K
SiO_2_	0	0	-	-	-
ZrO_2_	55	50	51	1.02	-
CuO/ZrO_2_	48	48	0	0	550
Al_2_O_3_	44	52	50	0.96	-
Ga_2_O_3_	0.1	2.2	2.1	0.95	-
CuO	5.8	4.7	1.7	0.36	620
NiO	0.6	2.0	1.3	0.65	710

## Data Availability

Not applicable.

## Sample Availability

Samples of the compounds are not available from the authors.

## References

[1] Spatari S., Zhang Y., MacLean H.L. (2005). Life cycle assessment of switchgrass- and corn stover-derived ethanol-fueled auto-mobiles. Environ. Sci. Technol..

[2] Farrell A.E., Plevin R.J., Turner B.T., Jones A.D., O’Hare M., Kammen D.M. (2006). Ethanol can contribute to energy and envi-ronmental goals. Science.

[3] Pimentel D., Patzek T.W. (2005). Ethanol Production Using Corn, Switchgrass, and wood; biodiesel production using soybean and sunflower. Nat. Resour. Res..

[4] Hou T., Zhang S., Chen Y., Wang D., Cai W. (2015). Hydrogen production from ethanol reforming: Catalysts and reaction mechanism. Renew. Sustain. Energy Rev..

[5] Akdim O., Cai W., Fierro V., Provendier H., Veen A., Shen W., Mirodatos C. (2008). Oxidative Steam Reforming of Ethanol over Ni–Cu/SiO_2_, Rh/Al_2_O_3_ and Ir/CeO_2_: Effect of Metal and Support on Reaction Mechanism. Top. Catal..

[6] Vargas P., Campos C.H., Navarro R.M., Fierro J.L.G., Reyes P. (2015). Rh/Al_2_O_3_–La_2_O_3_ catalysts promoted with CeO_2_ for ethanol steam reforming reaction. J. Mol. Catal. A Chem..

[7] Śliwa M., Socha P.R. (2022). Modification of CuO–ZrO_2_–ZnO Mixed Oxide Catalyst with Mn, Ga, Ni: Impact on Physicochemical Properties and Hydrogen Production via Low Temperature Steam Reforming of Ethanol. Catal. Lett..

[8] Roh H.S., Wang Y., King D.L., Platon A., Chin Y.H. (2006). Low Temperature and H_2_ Selective Catalysts for Ethanol Steam Reforming. Catal. Lett..

[9] Birot A., Epron F., Descorme C., Duprez D. (2008). Ethanol steam reforming over Rh/CexZr1−xO_2_ catalysts: Impact of the CO–CO_2_–CH_4_ interconversion reactions on the H_2_ production. Appl. Catal. B Environ..

[10] Koh A.C.W., Leong W.K., Chen L., Ang T.P., Lin J., Johnson B.F.G., Khimyak T. (2008). Highly efficient ruthenium and ruthenium–platinum cluster-derived nanocatalysts for hydrogen production via ethanol steam reforming. Catal. Commun..

[11] Wang F., Cai W.T., Provendier H., Schuurman Y., Descorme C., Mirodatos C., Shen W. (2012). Ageing analysis of a model Ir/CeO_2_ catalyst in ethanol steam reforming. Appl. Catal. B Environ..

[12] Goula M., Kontou S., Tsiakaras P. (2004). Hydrogen production by ethanol steam reforming over a commercial Pd/γ-Al_2_O_3_ catalyst. Appl. Catal. B Environ..

[13] Ciambelli P., Palma V., Ruggiero A. (2010). Low temperature catalytic steam reforming of ethanol. 1. The effect of the support on the activity and stability of Pt catalysts. Appl. Catal. B Environ..

[14] Song H., Ozkan U.S. (2010). Changing the Oxygen Mobility in Co/Ceria Catalysts by Ca Incorporation: Implications for Ethanol Steam Reforming. J. Phys. Chem. A.

[15] Frusteri F., Freni S., Spadaro L., Chiodo V., Bonura G., Donato S., Cavallaro S. (2004). H_2_ production for MC fuel cell by steam reforming of ethanol over MgO supported Pd, Rh, Ni and Co catalysts. Catal. Commun..

[16] Li S., Li M., Zhang C., Wang S., Ma X., Gong J. (2012). Steam reforming of ethanol over Ni/ZrO_2_ catalysts: Effect of support on product distribution. Int. J. Hydrogen Energy.

[17] Han S.J., Song J.H., Bang Y., Yoo J., Park S., Kang K.H., Song I.K. (2016). Hydrogen production by steam reforming of ethanol over mesoporous Cu–Ni–Al_2_O_3_–ZrO_2_ xerogel catalysts. Int. J. Hydrogen Energy.

[18] Garbarino G., Riani P., Lucchini M.A., Canepa F., Kawale S., Busca G. (2013). Cobalt-based nanoparticles as catalysts for low temperature hydrogen production by ethanol steam reforming. Int. J. Hydrogen Energy.

[19] Lorenzut B., Montini T., De Rogatisa L., Canton P., Benedetti A., Fornasiero P. (2011). Hydrogen production through alcohol steam reforming on Cu/ZnO-based catalysts. Appl. Catal. B Environ..

[20] Navarro R.M., Peña M.A., Fierro J.L.G. (2007). Hydrogen Production Reactions from Carbon Feedstocks: Fossil Fuels and Biomass. Chem. Rev..

[21] Galetti A.E., Gomez M.F., Arrua L.A., Marchi A.J., Abello M.C. (2008). Study of CuCoZnAl oxide as catalyst for the hydrogen production from ethanol reforming. Catal. Commun..

[22] Śliwa M., Samson K. (2021). Steam reforming of ethanol over copper-zirconia based catalysts doped with Mn, Ni, Ga. Int. J. Hydrogen Energy.

[23] Podobiński J., Zimowska M., Śliwa M., Datka J. (2023). IR Studies of Ethoxy Groups on CeO_2_. Molecules.

[24] Podobiński J., Śliwa M., Datka J. (2022). Ethoxy Groups on ZrO_2_, CuO, and CuO/ZrO_2_ Studied by IR Spectroscopy. Molecules.

[25] Binet C., Daturi M., Lavalley J.C. (1999). IR Study of polycrystalline ceria properties in oxidized and reduced states. Catal. Today.

[26] Badri A., Binet C., Lavalley J.C. (1996). An FTIR study of surface ceria hydroxy groups during a redox process with H_2_. J. Chem. Soc. Faraday Trans..

[27] Ma Z.Y., Yang C., Wei W., Li W.H., Sun Y.H. (2005). Surface properties and CO adsorption on zirconia polymorphs. J. Mol. Catal. A Chem..

[28] He M.Y., Ekerdt J.G. (1984). Infrared studies of the adsorption of synthesis gas on zirconium dioxide. J. Catal..

[29] Bensitel M., Saur O., Lavalley J.C., Mabilon G. (1987). Acidity of zirconium oxide and sulfated ZrO_2_ samples. Mater. Chem. Phys..

[30] Agron P.A., Fuller E.L., Holmes H.F. (1975). IR studies of water sorption on ZrO_2_ polymorphs. J. Colloid Interf. Sci..

[31] Tsyganenko A.A., Filimonov V.N. (1972). Infrared spectra of surface hydroxyl groups and crystalline structure of oxides. Spectrosc. Lett..

[32] Knözinger H. (1976). Specific Poisoning and Characterization of Catalytically Active Oxide Surfaces. Advances in Catalysis..

[33] West R., Baney R.H. (1960). The relationship between O-H stretching frequency and electronegativity in hydroxides of various elements. J. Phys. Chem..

[34] Peri J.B. (1971). Surface chemistry of AlPO_4_—a mixed oxide of Al and P. Discuss. Faraday Soc..

[35] Knözinger H., Ratnasamy P. (1978). Catalytic Aluminas: Surface Models and Characterization of Surface Sites. Catal. Rev. Sci. Eng..

[36] Arean C.O., Bellan A.L., Mentruit M.P., Delgado M.R., Palomino G.T. (2000). Preparation and characterization of mesoporous γ-Ga_2_O_3_. Microporous Mesoporous Mater..

[37] Cappus D., Xu C., Ehrlich D., Dillmann B., Ventrice C.A., Al Shamery K., Kuhlenbeck H., Freud H.J. (1993). Hydroxyl groups on oxide surfaces: NiO (100), NiO (111), and Cr_2_O_3_ (111). Chem. Physic.

[38] Yee A., Morrison S.J., Idriss H. (1999). A Study of the Reactions of Ethanol on CeO_2_ and Pd/CeO_2_ by Steady State Reactions, Temperature Programmed Desorption, and In Situ FT-IR. J. Catal..

[39] Ito K., Bernstein H.J. (1956). The Vibrational Spectra of Formate, Acetate and Oxalate Ions. Can. J. Chem..

